# Evaluation of a Computer-Based Training Program for Enhancing Arithmetic Skills and Spatial Number Representation in Primary School Children

**DOI:** 10.3389/fpsyg.2016.00913

**Published:** 2016-06-27

**Authors:** Larissa Rauscher, Juliane Kohn, Tanja Käser, Verena Mayer, Karin Kucian, Ursina McCaskey, Günter Esser, Michael von Aster

**Affiliations:** ^1^Department of Psychology, University of PotsdamPotsdam, Germany; ^2^Computer Graphics Laboratory, ETH ZurichZurich, Switzerland; ^3^Children's Research Center, University Children's HospitalZurich, Switzerland; ^4^MR-Center, University Children's HospitalZurich, Switzerland; ^5^Department of Child and Adolescent Psychiatry, German Red Cross HospitalsBerlin, Germany

**Keywords:** numerical development, evaluative study, primary school, computer-based training, mathematics instruction

## Abstract

Calcularis is a computer-based training program which focuses on basic numerical skills, spatial representation of numbers and arithmetic operations. The program includes a user model allowing flexible adaptation to the child's individual knowledge and learning profile. The study design to evaluate the training comprises three conditions (Calcularis group, waiting control group, spelling training group). One hundred and thirty-eight children from second to fifth grade participated in the study. Training duration comprised a minimum of 24 training sessions of 20 min within a time period of 6–8 weeks. Compared to the group without training (waiting control group) and the group with an alternative training (spelling training group), the children of the Calcularis group demonstrated a higher benefit in subtraction and number line estimation with medium to large effect sizes. Therefore, Calcularis can be used effectively to support children in arithmetic performance and spatial number representation.

## Introduction

Already at an early stage of development there are considerable differences between children regarding number processing and calculation (Dowker, 2005). Difficulties and deficits that occur at an early learning stage potentially have an adverse impact on the further learning course and are predictive for “failure to progress” (Jordan et al., 2003; Morgan et al., 2009). Especially basic numerical skills have a highly predictive value for future learning process (Kaufmann et al., 2005; Jordan et al., 2007; Krajewski and Schneider, 2009) and early differences in basic numerical skills tend to become more pronounced with increasing age during primary school (Aunola et al., 2004; Geary, 2006). In addition to basic numerical skills, findings show that the mastery of basic arithmetical operations is substantial for the further development of mathematical abilities in primary school (Mercer and Miller, 1992; Van Luit and Naglieri, 1999; Duncan et al., 2007). These findings demonstrate the relevance of targeted interventions which ensure that basic math skills are sufficiently developed at an early stage to establish a solid foundation for future learning processes. This study aims to evaluate an adaptive program built on theoretical models of number processing and numerical development. It offers children at an early stage the possibility to train exactly those aspects of number processing and arithmetic skills, in which they still need support.

### Theoretical models of number processing and numerical development

In the past years, different models of number processing and calculation have been proposed (e.g., McCloskey et al., 1985; Cipolotti and Butterworth, 1995). The triple-code model (Dehaene, 1992) is currently the most influential model. It presumes an integrative network, which is specified by three different modules (verbal, Arabic, and analog magnitude) related to number processing. The modules are characterized by different representational properties and functions of numbers and are connected by bidirectional transcoding links. The verbal module supports counting and number fact retrieval, the visual-Arabic module is required for solving written arithmetic and the analog magnitude module for semantic number processing. Dehaene et al. (1999) showed that the basic components of mathematical development can be allocated to specific areas of the brain using functional neuroimaging. Concerning estimation and exact calculation the authors identified different brain areas involved during processing. These findings support the assumptions of an “analog representation of magnitudes” and an “auditory-verbal representation” (Jacobs and Petermann, 2003). Based on their fMRI meta-analysis, Arsalidou and Taylor (2011) suggested an extension of the triple-code model. The authors demonstrated the relevance of supporting and domain-general functions involved in solving arithmetic tasks. Furthermore, recent research indicates that the overlap of number representations across different number notations increases with age, expertise and schooling (Kucian and Kaufmann, 2009; Kaufmann et al., 2011). Other fMRI studies indicate that the mental number line emerges during the first school years in the parietal lobe as a result of practice and experience (Rivera et al., 2005; Kucian et al., 2008).

The triple-code model constitutes the end state of numerical development and calculation abilities. Several theoretical models address the question how numerical cognition develops over childhood (for example, von Aster, 2005; Krajewski and Schneider, 2009; Kaufmann et al., 2011). The developmental model of Krajewski (2008) describes how the ability of counting is linked with quantities and quantity operations and how quantity-number-competencies are acquired over three levels. However, this model does not depict the entire developmental process of numerical cognition, as it is limited to primary school age. The four-step developmental model (von Aster and Shalev, 2007) is based on recent findings of developmental psychology and cognitive sciences. It assumes the development of different cognitive representations (cardinality, verbal number system, Arabic number system, ordinality) relevant for number processing and calculation as a neuroplastic process, which is intertwined with the complementary advancement of other cognitive domains and domain-general abilities such as attention or working memory (von Aster and Shalev, 2007; Kaufmann and von Aster, 2012). According to this model the inherited core-system representation of cardinal magnitude (step 1), which allows functions such as subitizing and approximating, provides the basic meaning of numbers. The next step includes the learning process of associating a perceived number to a spoken symbol internalizing verbal systems (number words, step 2) and later Arabic systems (digits, step 3). The symbolization starts with the beginning of language development. The internalization of these systems is the precondition for the formation of abstract spatial number representation (mental number line) (step 4) in primary school, which has been found to be essential for arithmetic thinking (von Aster, 2005). This mental number line is assumed to develop in parallel to the acquisition of the symbolization systems and the resulting growing number of arithmetic operations. The described processes in this model cannot be considered in isolation from the development of other cognitive abilities, such as writing and reading or domain-general functions, such as working memory or attention. Furthermore, the development of the different modules depends on experience and is the product of an individual and socio-culturally influenced learning history. According to this model, delays or deficits in the development of early abilities and functions result in difficulties in constructing a spatial representation of numbers, regardless whether the child has deficits in basic numerical abilities or deficits in mapping the symbolization systems (verbal and/or arabic) (von Aster, 2005). A major benefit of this model is that typical development as well as different pathways of pathological development can be mapped (von Aster and Shalev, 2007). Furthermore, the model enables predictions of etiologic constellations and possible neuropsychological dysfunctions for developmental dyscalculia (von Aster et al., 2007). Delays and deficits in the development of early abilities and functions can occur at different stages leading to individual learning needs (von Aster, 2005). Moreover, it becomes apparent that training approaches that focus only on one aspect, e.g., training of basic numerical abilities or the repeated practice of arithmetic operations, do not meet the challenges of this multilayered process. An effective training approach requires a course of action in which the hierarchically organized and partly in parallel proceeding processes of the postulated steps are trained according to the individual profile of ability and knowledge.

### Computer-based interventions to enhance number processing and arithmetic skills

Designing a program to enhance number processing and arithmetic skills involves the consideration of a series of challenging aspects. Children differ with respect to learning speed (Brown et al., 2003) and benefit to differing degrees from practice (Schoppek and Tulis, 2010), which leads to various mathematical performance and deficit profiles (von Aster, 2000; Geary, 2004; Wilson and Dehaene, 2007). Furthermore, children enter formal school with highly diverse performance preconditions (Hair et al., 2006). To respond adequately to these factors a considerable amount of individualization is necessary.

A computer-based training adapting to the child's individual learning profile and development level can contribute to these requirements. It allows an optimal level of difficulty and learning speed by an individually customized task selection. Another key advantage is the possibility of immediate feedback about the correctness of a solved task. Direct chronological proximity is crucial for knowledge acquisition (Krajewski and Ennemoser, 2010) and allows a correct modeling of the task solution.

Furthermore, the computer represents an attractive learning medium (Kulik and Kulik, 1991; Schoppek and Tulis, 2010) providing intensive training in a stimulating environment (Kulik, 2004). Computerized learning programs can be designed to be particularly suitable for children and are rather associated with playing than with learning (Lenhard et al., 2011).

Particularly for children experiencing difficulties in math, a computerized training provides the possibility of a learning environment detached from performance pressure and peer comparisons in school context and offers therefore a less stressful and risk-free setting to explore mathematics (Käser and von Aster, 2013).

### Efficacy of computerized training programs

Different meta-analyses examined the effects of computer-based math instruction, revealing positive effects. For example, Kulik (Kulik and Kulik, 1991; Kulik, 1994) reported an average effect size of 0.47 for computer-based math instruction in elementary school. Li and Ma (2010) specified that particularly students with special needs benefit from computer-based instruction and that larger effects are found for elementary school than for higher education. Likewise, other studies revealed positive effects for computer-based math instruction with effect sizes ranging from 0.13 to 0.8 (Khalili and Shashaani, 1994; Fletcher-Flinn and Gravatt, 1995; Kroesbergen and van Luit, 2003; Slavin and Lake, 2008; Ise et al., 2012; Chodura et al., 2015). Beside these promising effects, studies comparing the effectiveness of computer-based programs with other instructional methods for mathematics reported that computer-based instruction is less effective than direct teacher instruction (Kroesbergen and van Luit, 2003; Ise et al., 2012). However, a recent meta-analysis conducted by Chodura et al. (2015) revealed that computer-based interventions are as effective as interventions with human trainers.

Although a number of different computerized learning programs for mathematics exist, the majority of the programs lack empirically based analyses of their effectiveness (Butterworth and Laurillard, 2010; Butterworth et al., 2011) or a theoretically substantiated basis. There are computer-based programs, which have been shown to be effective in enhancing number processing or arithmetic fact knowledge (Fuchs et al., 2006; Wilson et al., 2006; Lenhard and Lenhard, 2010; Kucian et al., 2011), but the available programs mostly focus on specific skills and offer only limited adaptability. Furthermore, evaluative studies include only small sample sizes (Räsänen et al., 2009) and study designs include solely the comparison to an untrained control group (Räsänen et al., 2009; Lenhard and Lenhard, 2010; Kucian et al., 2011) whereas comparisons to groups receiving alternative trainings are missing.

In the following, a selection of empirically evaluated computerized trainings is presented. “Rechenspiele mit Elfe und Mathis I” (Lenhard and Lenhard, 2010) is a math training program for children of the first to third grade on the basis of the national educational standards. The program consists of five components (quantities, numbers, geometry, word problems, arithmetic), which refer to content areas of math class. The evaluative study revealed a positive effect of the training on the children's mathematics achievement in a standardized test compared to a control condition of children attending regular math class. However, it should be noted that the controlling of the task sequences and games does not follow a theory-based hierarchy of learning objectives, which impedes the adaption to the child‘s individual learning difficulties (Käser and von Aster, 2013).

The program “Number Race” (Wilson et al., 2006) builds on the assumption, that dyscalculia results from a core deficit in number sense or deficits concerning the link between number sense and symbolic number representations. The adaptive software is designed to train numerical comparisons and enhance quantity representation. The evaluation of the program with a small sample of children aged 7–9 years with mathematical learning difficulties demonstrated significant improvements in basic numerical cognition, but the effects did not generalize to counting or arithmetic (Wilson et al., 2006). Räsänen et al. (2009) compared “Number Race” to a game (Graphogame-Math; Mönkkönen et al., in preparation) which trains the matching of verbal labels to visual patterns and number symbols of small sets of exact numerosities. In this study, kindergarten children with low numeracy skills (*n* = 30) were randomly assigned to one of the two training conditions. Children trained on a daily basis for 3 weeks. Compared to a group of typically performing children (*n* = 30), both training groups demonstrated improved skills in number comparison, but not in other areas of number skills (verbal counting, number comparison, object counting, arithmetic).

The objective of the program “Rescue Calcularis” (Kucian et al., 2011) is to improve the construction of and access to the mental number line. Basic aspects of number processing as well as addition and subtraction are trained. Children are presented with an Arabic digit, an addition/subtraction problem or a number of dots and the challenge is to position the result of each task on the number line using a joystick. The evaluation of the program revealed positive effects for children with and without developmental dyscalculia as measured with neuropsychological tests and functional magnetic resonance imaging (fMRI) (Kucian et al., 2011). Children showed an improved spatial representation of numbers as well as improved arithmetical performance. Furthermore, fMRI analyses revealed that both groups showed reduced recruitment of brain regions supporting number processing after the training, which can be attributed to a modulation of neural activation, that facilitates processing of numerical tasks.

Calcularis uses core elements of “Rescue Calcularis” (Kucian et al., 2011), but represents a more complete training of mathematical skills and employs a user model allowing flexible adaptation. As in “Rescue Calcularis,” the program Calcularis places a special focus on the creation of and access to the mental number line. However, it is extended by training elements to automatize the different number representations and their interrelations and arithmetic operations in expanding number ranges. Calcularis was evaluated in a pilot study with a small sample (*N* = 32) of Swiss children with difficulties in learning mathematics (Käser et al., 2013). Children benefited significantly from the training regarding number representation and subtraction.

The present article represents the evaluation of Calcularis in a large study sample (*N* = 138) using a study design with two control groups.

### Research aims

The main objective of the present study is to evaluate the efficacy of the computer-based training program Calcularis by combining two different approaches. First, we aimed to determine the general immediate efficacy by comparing the Calcularis training group with an untrained control group. The implementation of an untrained waiting control group allows controlling for developmental and schooling effects as well as arithmetic development under regular conditions. Second, we compared the performance of the Calcularis training group with a group that received a computerized spelling training to examine the domain specificity of the training effects. Thus, the efficacy of the training can be determined by taking novelty and Hawthorne effects as well as unspecific training effects on domain-general functions into consideration. We hypothesized that the Calcularis group will demonstrate an increased arithmetic performance (addition, subtraction) and spatial number representation (in the number range 0–10 and 0–100) compared to both groups with small (computerized spelling training) to medium (untrained control group) effect sizes.

## Materials and methods

### Introduction to calcularis

**Figure 1:**
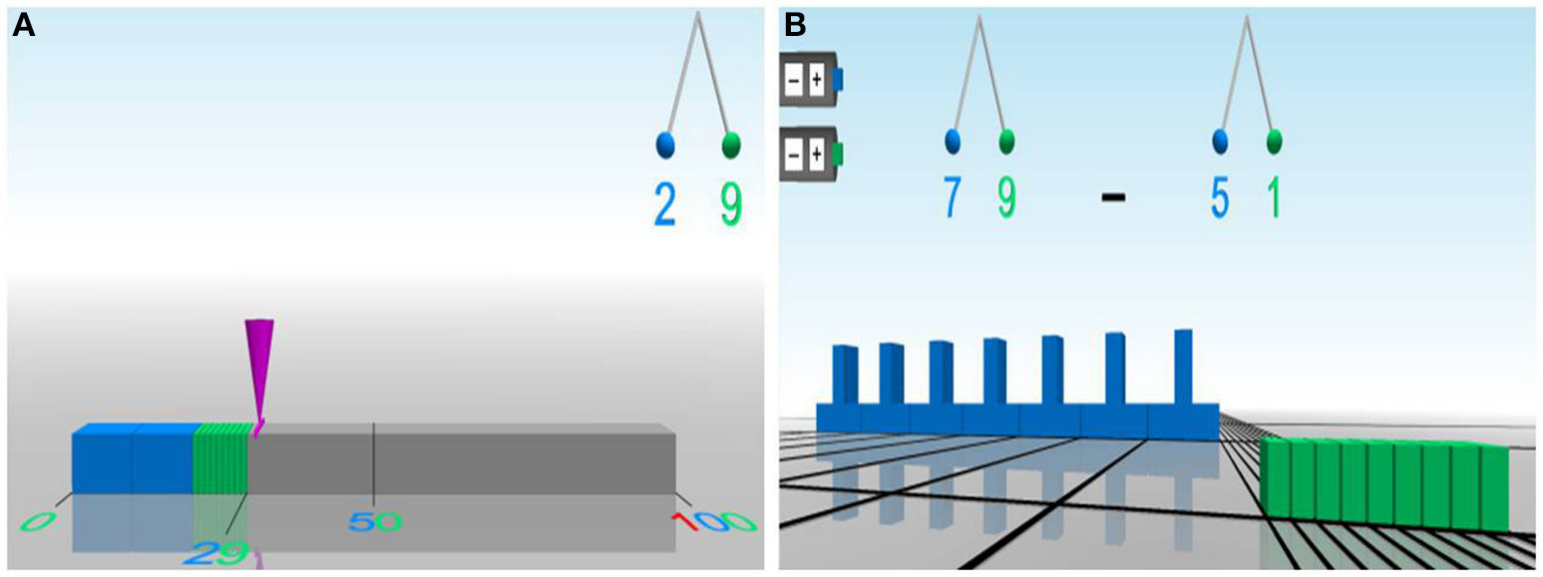
**Screenshots from the computer-based training program Calcularis**. In the LANDING game **(A)**, the position of the displayed number (29) needs to be indicated on the number line. In the PLUS-MINUS game **(B)**, the task displayed needs to be modeled with the blocks of tens and ones.

Calcularis is an adaptive computer-based training program. The program‘s theoretical foundation of numerical cognition and development is based on the triple-code model (Dehaene, 1992) and the four-step developmental model (von Aster, 2005; von Aster and Shalev, 2007). It aims to automatize the different number representations and their interrelations, support the formation of and access to a mental number line and practice arithmetic operations as well as arithmetic fact knowledge in expanding number ranges. Thus, the training covers a wide range of aspects of numerical cognition which increases the programs adaptability to the individual child's deficits and learning needs. Calcularis consists of different instructional games, which are hierarchically structured according to number ranges and can be further divided into two areas. The first area focuses on different number representations as well as number processing in general. Transcoding between alternative representations is trained and children learn the three principles of number understanding: cardinality, ordinality, and relativity. Games in this area are hierarchically ordered according to the four-step developmental model (von Aster and Shalev, 2007). The first area is exemplified by the LANDING game illustrated in Figure 1A. In this game, children need to indicate the position of a given number on a number line. To do so, a falling cone has to be steered using a joystick. The second area covers cognitive operations and procedures with numbers. In this area, children train on the concepts and automation of arithmetic operations. The difficulty of the tasks is determined by task complexity, the magnitude of numbers involved and the visual aids available to solve the task. For example, in the PLUS-MINUS game (see Figure 1B), children solve addition and subtraction tasks using blocks of tens and ones.

The key components (number representation, arithmetic operations) are trained by main and support games. While main games require a combination of abilities, support games train specific skills that serve as prerequisites for the main games. A consistent number notation that accentuates the properties of numbers is used throughout the training program. The notation is encoded by color, form and topology. It is assumed, that this design of the numerical stimuli enhances the different number modalities and strengthens the link between them. A more detailed description of the training including a selection of examples of the games can be found in Käser et al. (2013) as well as in Räsänen et al. (2015).

Calcularis includes a user model allowing flexible adaptation on the basis of the internally mapped learning and knowledge profile of the individual child. All children start the training with the same game. After each completed item, the program estimates the actual knowledge state of the child and displays a new task adjusted to this state. The mathematical structure of Calcularis represents a model of the cognitive processes of mathematical development by a dynamic Bayes net. The Bayes net comprises a directed acyclic graph which represents various mathematical skills and their relationships. The user model is based on the student model and control algorithm presented in Käser et al. (2012). Furthermore, repetitions of mastered skills (e.g., subitizing or arithmetic operations in already mastered number ranges) are implemented to strengthen trained abilities. A bug library with typical error patterns allows to provide targeted games for the remediation of specific errors.

### Instruments

#### Basic diagnostics of specific developmental disorders in elementary school age children (BUEGA)

The BUEGA (Esser et al., 2008) served for the assessment of verbal and nonverbal intelligence as well as the performance in reading, writing and arithmetic. Reading performance is assessed via reading speed and reading accuracy. Writing performance is evaluated by the correct writing of words/ graphemes. The subscale “arithmetic performance” assesses the performance in the four basic arithmetical operations (addition, subtraction, multiplication, and division) as the child solves a series of math word problems. The internal consistency coefficients determined for each school grade are sufficient to high (α = 0.81 to α = 0.95).

#### HAWIK-IV (Hamburg-Wechsler intelligence test for children)

Two subtests (similarities, block design) of the HAWIK-IV (Hamburg-Wechsler intelligence test for children; Petermann and Petermann, 2007), the German version of the WISC-IV (Wechsler Intelligence Scale for Children; Wechsler, 2003) were used to measure verbal and nonverbal intelligence. Both subtests show good psychometric properties for children aged 7 to 11 years with reliabilities of *r* = 0.84–0.88 for block design and *r* = 0.85–0.89 for similarities (split-half coefficients, Spearman-Brown corrected).

#### Heidelberger rechentest 1–4 (HRT)

Arithmetic performance was assessed on the basis of the two subscales “addition” and “subtraction” of the HRT (Haffner et al., 2005). The HRT is designed as a speed test and addresses specifically computational fluency. A list of 40 addition/subtraction tasks is presented to the children with the goal to solve as many problems as possible within 2 min. As an index of reliability, retest reliability was calculated over a 2-week period with high coefficients for addition (*r*_*tt*_ = 0.82) and subtraction (*r*_*tt*_ = 0.86).

#### Arithmetic performance test

The arithmetic performance test (Kucian et al., 2011) served as a power test examining arithmetic performance within a number range up to 100. The children are presented with 20 addition/subtraction tasks without a time limit. As a power test this arithmetic performance test aims to compute the children's degree of mastery of addition and subtraction under conditions of zero time pressure.

#### Number line test

As a measure of spatial representation of numbers, children indicated the location of 20 verbally and visually presented numbers on a number line from 0 to 100. The items of the number line test were evenly distributed across the number range from 0 to 100 as two numbers of every teen were selected. The percent absolute estimation error (PAE) for target number and the indicated location (estimated number) on the number line was calculated (PAE = |estimated number – target number|/scale of estimates, cf. Siegler and Booth, 2004). In addition, to evaluate the linearity of the spatial representation we calculated the correlation coefficient of linear fit (*R*^2^ lin) for each child.

#### Computer-test

A computer-based mathematical test (Käser et al., 2013) was conducted that assessed arithmetical performance and spatial representation of numbers. This speed-test includes subtests for addition/subtraction with a number range from 0 to 1000. Children are asked to solve addition/subtraction tasks of increasing difficulty within a time period of 10 min. The maximum number of items is 76 for each subtest.

The subtest for *spatial representation of numbers* comprises a number line test with a number range of 0–10. A number is presented verbally and visually on the screen and children are asked to indicate the position on a number line by mouse-click. In total, the subtest includes 10 items. We calculated the percent absolute estimation error (PAE) as well as *R*^2^ lin of the individual number line estimation (NLE) pattern.

We used three different tests (HRT, arithmetic test and computer-based mathematical test) examining the performance in addition and subtraction tasks. These instruments were implemented since they focus on specific aspects (e.g., computational fluency, degree of mastery) of arithmetic in different number ranges. Furthermore, they are used to compare the results to those of previous studies examining the efficacy of computer based trainings on spatial number representation and calculation (Kucian et al., 2011; Käser et al., 2013). To provide high comparability to previous findings of Käser et al. (2013) with regard to spatial number processing two number line task with different number ranges (0–10, 0–100) were implemented.

#### Feedback questionnaire

Children and their parents completed a training evaluation questionnaire at the end of the study. The questionnaire includes 4 items concerning the self-evaluation of the enjoyment of the training, improvement of self-perceived arithmetic skills and changes regarding self-confidence. For example, children were presented with the statement “I enjoyed the training” and responded on a 4-point Likert scale ranging from “disagree” to “strongly agree” (0–3). The internal consistency coefficients determined for the children that trained with Calcularis is satisfactory (α = 0.83). Additionally, children judged the difficulty level of the training on a 5-point Likert scale ranging from “far too simple” to “much too difficult.” Furthermore, parents were asked to rate how much their child liked the training (“My child enjoyed the training”).

### Study design and sample

The study design comprised three groups (Calcularis group, waiting group, spelling training group). Children were randomly assigned to one of three groups. The Calcularis group completed a 6–8 weeks training whilst the waiting group started the training with a 6 week rest period. The spelling training group served as a second control group receiving a computer-based spelling training (Dybuster; Kast et al., 2007) with the same training duration and frequency of training sessions as Calcularis. Dybuster is a computer-based training program designed for enhancing spelling skills. Evaluative studies of the program demonstrated that dyslexic children as well as typically achieving children improved their spelling skills (Kast et al., 2007, 2011).

Children trained with the program 5 times per week with daily training sessions of 20 min in their own home environment. Initial diagnostic (*t*_1_) included the assessment of arithmetic performance (BUEGA, HRT, arithmetic performance test, computer-test) as well as intelligence (HAWIK-IV, BUEGA), reading and spelling (BUEGA), and spatial representation of numbers (number line test, computer-test). All measures for arithmetic performance and spatial representation of numbers, except for the BUEGA and HAWIK-IV, were re-assessed at the second measurement point (*t*_2_).

Children were recruited via flyers sent to elementary schools and psychotherapeutic outpatient clinics. All children attended regular primary schools. Inclusion criteria comprised at least average IQ-scores (min: 16th percentile, T-score ≥ 40) (BUEGA, HAWIK-IV) and age 7;0 to 10;11 years.

One hundred and fifty-five German-speaking children were included in the study. Children were randomly assigned to the groups (Calcularis group: *n* = 54, waiting group: *n* = 50, spelling training group: *n* = 51). Only children with at least 24 sessions Calcularis/ Dybuster within a maximum of 10 weeks training period were included in data analyses. Owing to these inclusion criteria as well as other reasons such as illness during the training or test session, 11 children from the Calcularis group, 1 child from the waiting group and 5 children of the spelling training group were excluded.

### Statistical analyses

Group differences were analyzed by means of Analyses of Variance (ANOVA) and Chi-square tests. A series of repeated measures general linear model (GLM) analyses were conducted to evaluate training effects with assessment time point (*t*_1_–*t*_2_) as a within-subject factor and group (Calcularis/waiting/spelling training) as a between-subject factor. The main effects of group or time (over-all) are reported. The group × time interaction was the primary effect of interest. In case of a significant overall group × time interaction (comparison of all three groups), further GLM were calculated to determine the interaction effects of the pairs of groups (Calcularis vs. waiting/Calcularis vs. spelling training). Effect sizes are expressed as partial η^2^ coefficients. Cohen (1988) postulated that η^2^ values between 0.06 and 0.13 are medium effects and η^2^ values greater than 0.14 are large effects.

## Results

The final study sample consisted of 138 children at the age of 7;0 to 10;11. The mean age was 8.46 (*SD* = 0.79) years. The majority of children attended the second (*n* = 69, 50%) and third (*n* = 53, 38.4%) grade. There were 13 fourth graders (9.4%) and only three fifth graders (2.2%). The study population involved more girls (*n* = 95) than boys (*n* = 43). Children trained with the program for an average training duration of 6.95 (*SD* = 1.23, min = 4.71, max = 9.86) weeks and completed on average 29.57 (*SD* = 1.75, min = 25, max = 35) training sessions. Statistical analyses revealed no significant differences between the three groups for gender, age, arithmetic/ numerical performance or control variables (intelligence, writing, reading) in the initial diagnostic procedure (*t*_1_) (see Table 1). Analyses regarding the mean intelligence and arithmetic scores demonstrated a high variance within the sample (IQ mean T-score: 50.13, *SD* = 5.38, min = 41.33, max = 73.42; math mean T-score: 41.90, *SD* = 10.22, min = 21.67, max = 71.00).

**Table 1:** **Demographic and cognitive (control) characteristics [Means, (*SD*)], of the Calcularis group (CAL), the waiting group (WG) and the spelling training group (ST) prior to intervention**.

	**CAL (*n* = 43)**	**WG (*n* = 49)**	**ST (*n* = 46)**	**Test statistic**	***p***
Age (years)	8.48 (0.86)	8.54 (0.84)	8.34 (0.66)	0.82^b^	0.444
Gender (f/m)	31/12	31/18	33/13	1.10^c^	0.576
BUEGA verbal intelligence^a^	50.02 (8.49)	49.14 (9.90)	48.02 (9.03)	0.53^b^	0.589
BUEGA nonverbal intelligence^a^	53.40 (8.61)	51.24 (8.68)	53.70 (9.13)	1.09^b^	0.338
BUEGA reading^a^	49.98 (11.10)	49.16 (12.15)	46.91 (10.01)	0.92^b^	0.403
BUEGA writing^a^	44.93 (12.40)	45.12 (12.26)	44.22 (12.15)	0.07^b^	0.934
HAWIK IV block design^a^	49.38 (7.84)	46.74 (7.00)	47.75 (7.83)	1.54^b^	0.218
HAWIK IV similarities^a^	51.78 (8.49)	49.30 (6.65)	51.23 (6.97)	1.06^b^	0.351
Mean intelligence (HAWIK, BUEGA)	51.15 (5.86)	49.18 (4.94)	50.18 (5.33)	1.54^b^	0.219
BUEGA math word problems^a^	44.23 (12.24)	44.73 (12.11)	45.22 (12.50)	0.07^b^	0.931
Mean arithmetic performance^a^ (BUEGA, HRT addition, HRT subtraction)	41.28 (10.96)	42.16 (10.03)	42.22 (9.88)	0.12^b^	0.891

a *T-score*,

b *F-score*,

c *χ^2^-score*.

Using the highest level of education of either parent as an index of socioeconomic status (SES), the results demonstrated that 44% finished university (*n* = 55), 21% qualified to study at university (*n* = 26), 32% (*n* = 39) acquired the Mittlere Reife [qualification awarded after 10 years of secondary school] and 3 % (*n* = 4) completed Hauptschule [lower secondary education]. The data concerning the parent's educational level was missing for 14 children. There were no significant differences for the level of parental education between the three groups using chi-square test [$\chi_{\left(6\right)}^{2}=8.57$, *p* = 0.200].

**Table 2** summarizes the mean values and standard deviations of the mathematical performance measures before (*t*_1_) and after training or waiting period (*t*_2_) for the three groups.

### HRT

The repeated-measures GLM for the HRT addition task demonstrated a significant main effect of time [*F*_(1, 135)_ = 29.34, *p* < 0.001, *p* = 0.028], but no main effect of group [*F*_(2, 135)_ = 0.062, *p* = 0.940]. The group × time interaction was also not significant [*F*_(2, 135)_ = 1.92, *p* = 0.150].

For HRT subtraction, results demonstrated a significant main effect of time [*F*_(1, 135)_ = 27.01, *p* < 0.001, η^2^ = 167], but no main effect of group [*F*_(2, 135)_ = 0.11, *p* = 0.895]. The group × time interaction reached significant values [*F*_(2, 135)_ = 7.94, *p* = 0.001, η^2^ = 0.110], indicating that training progress differed between groups over time. Further analyses revealed that the children of the Calcularis group showed a higher increase in performance than the children of the waiting group [*F*_(1, 90)_ = 11.07, *p* = 0.001, η^2^ = 0.110] and the spelling training group with moderate effect sizes [*F*_(1, 87)_ = 13.64, *p* = 0.001, η^2^ = 0.136].

### Arithmetic performance test

The GLM revealed a significant main effect of time [*F*_(1, 135)_ = 6.89, *p* = 0.01], but no main effect of group [*F*_(2, 135)_ = 0.83, *p* = 0.438] for addition. The group × time interaction was also not significant [*F*_(1, 135)_ = 1.77, *p* = 0.173].

For subtraction, results demonstrated a significant main effect of time [*F*_(1, 135)_ = 13.34, *p* < 0.001, η^2^ = 0.09], but no main effect of group [*F*_(2, 135)_ = 0.21, *p* = 0.813]. There was a significant group × time interaction [*F*_(2, 135)_ = 4.27, *p* = 0.016, η^2^ = 0.06]. Subsequent analyses revealed that the children of the Calcularis group demonstrated a significantly larger improvement in performance than the children of the waiting group [*F*_(1, 90)_ = 8.88, *p* = 0.001, η^2^ = 0.09, medium effect size] and the spelling training group [*F*_(1, 87)_ = 4.14, *p* = 0.045, η^2^ = 0.045, small effect size].

### Number line test

#### Percent absolute error (PAE)

The analysis for the number line test yielded no main effects of time [*F*_(1, 135)_ = 1.40, *p* = 0.239] and group [*F*_(2, 135)_ = 0.81, *p* = 0.445]. The group × time interaction was significant [*F*_(2, 135)_ = 3.99, *p* = 0.021, η^2^ = 0.056]. The children of the Calcularis group showed a higher gain compared to the children of the spelling group with medium effect size [*F*_(1, 87)_ = 6.21, *p* = 0.015, η^2^ = 0.067]. The interaction between group × time was not significant for the comparison between the Calcularis and the waiting group [*F*_(1, 90)_ = 3.42, *p* = 0.068].

#### Linearity

**Figure 2:**
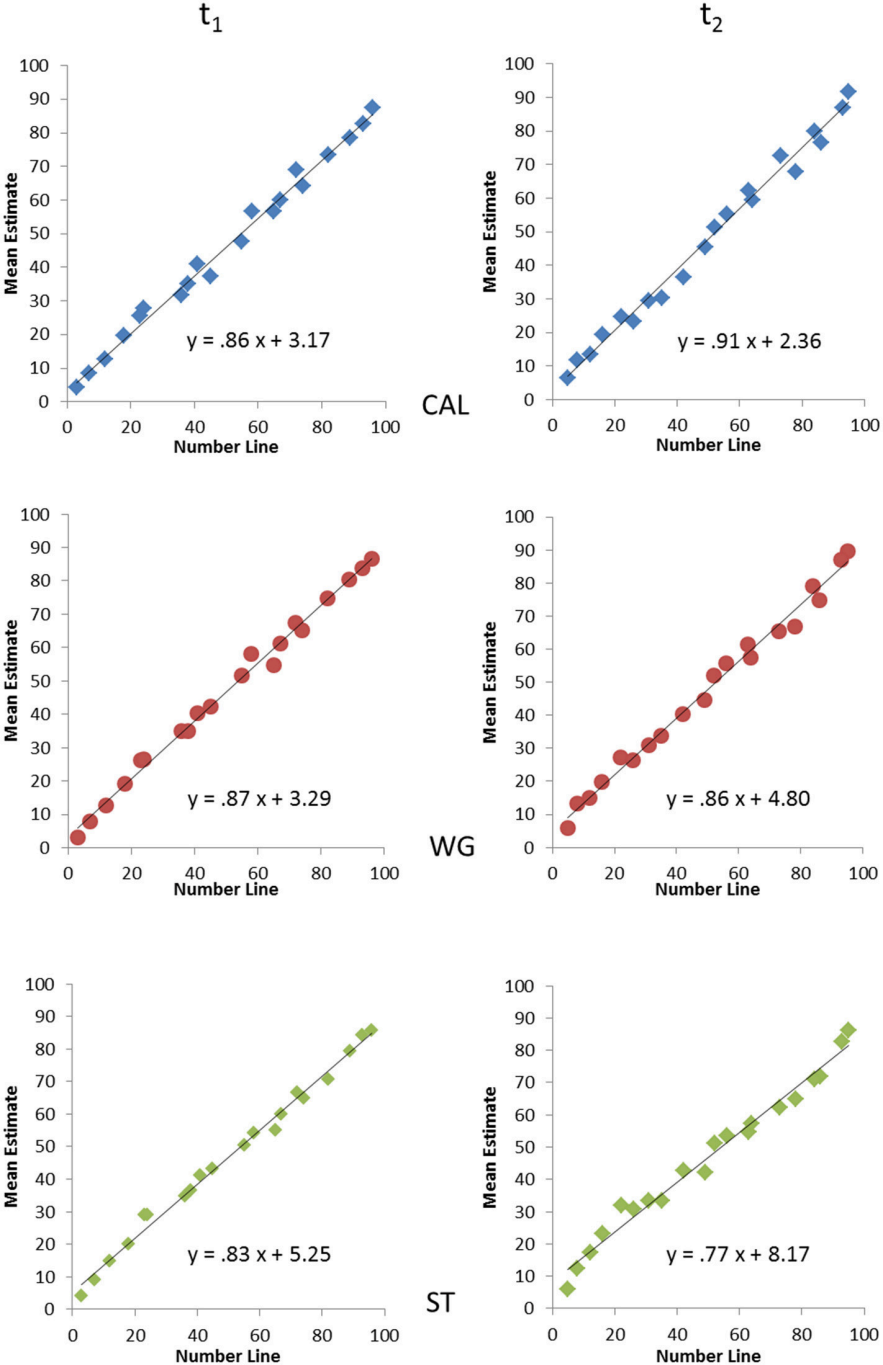
**Estimation patterns for the number line test 0–100 for the three groups**. Regression functions and correlation coefficients for the linear fit (mean estimates calculated for each group separately) are shown before (left column) and after training or waiting period (right column).

The initial analyses examined whether the spatial representation is better explained by a linear or logarithmic function. The regressions to the estimates of children for each of the 20 numbers that were presented were calculated for each child. A paired-sample *t*-test comparing the mean absolute value of residuals of the linear and of the logarithmic fit for each child was performed. Results indicate that the spatial representation of numbers is better explained by a linear than a logarithmic fit [*t*_(137)_ = −16, 97, *p* < 0.001], since residuals are smaller for linear than for logarithmic fit. Therefore, we used *R*^2^ lin of each child for the GLM. To get an impression of the fit of each group mean estimates were calculated separately for each training group and plotted as a function of target number (Figure 2).

The analysis regarding individual *R*^2^ lin (see Table 2) indicated that there was no main effect of time [*F*_(1, 135)_ = 0.20, *p* = 0.667] and no main effect of group [*F*_(2, 135)_ = 0.64, *p* = 0.531]. The group × time interaction was not significant [*F*_(2, 135)_ = 1.12, *p* = 0.331].

**Table 2:** **Training effects (mean values and standard deviations) of the Calcularis group (CAL), waiting group (WG) and spelling training group (ST) on arithmetic performance and spatial number representation**.

**Outcome parameter**	**Group**	***n***	***t*_1_*M* (*SD*)**	***t*_2_*M* (*SD*)**
HRT (addition)^a^	CAL	43	39.91 (11.03)	43.70 (11.18)
	WG	49	40.69 (10.73)	43.73 (11.74)
	ST	46	40.78 (9.58)	42.17 (9.56)
HRT (subtraction)^a^	CAL	43	39.70 (11.22)	44.70 (11.37)
	WG	49	41.04 (10.96)	42.24 (12.23)
	ST	46	40.65 (10.30)	41.59 (10.42)
Arithmetic performance test (addition)^b^	CAL	43	15.67 (4.61)	17.00 (3.94)
	WG	49	15.37 (5.60)	15.65 (4.80)
	ST	46	14.93 (4.73)	15.28 (4.85)
Arithmetic performance test (subtraction)^b^	CAL	43	13.12 (4.81)	14.79 (4.02)
	WG	49	13.45 (5.12)	13.61 (5.61)
	ST	46	13.02 (5.13)	13.57 (5.30)
Number line test, PAE^c^	CAL	43	9.13 (4.26)	7.90 (5.02)
	WG	49	8.27 (3.47)	8.03 (4.24)
	ST	46	8.94 (4.53)	9.51 (4.94)
Number line test, linearity^d^	CAL	43	0.86 (0.16)	0.87 (0.20)
	WG	49	0.88 (0.13)	0.88 (0.15)
	ST	46	0.85 (0.19)	0.82 (0.21)
Computer-test (addition)^b^	CAL	33	28.03 (7.94)	29.88 (12.55)
	WG	35	33.69 (15.51)	32.29 (14.38)
	ST	35	27.51 (11.41)	28.83 (12.36)
Computer-test (subtraction)^b^	CAL	33	21.58 (9.48)	25.46 (11.84)
	WG	35	27.17 (12.76)	25.09 (12.05)
	ST	35	22.60 (11.20)	23.83 (11.78)
Computer-test (number line 0-10), PAE^c^	CAL	29	1.40 (0.97)	0.81 (0.86)
	WG	32	0.80 (0.51)	1.01 (0.89)
	ST	29	1.29 (0.95)	1.37 (0.99)
Computer-test (number line 0-10), linearity^d^	CAL	29	0.82 (0.20)	0.93 (0.08)
	WG	32	0.90 (0.12)	0.86 (0.21)
	ST	29	0.86 (0.17)	0.79 (0.24)

a *T-score*.

b *Number of correctly solved items*.

c *Percent absolute error*.

d *R^2^ lin*.

### Computer-test

#### Addition and subtraction

The analyses for the addition task did not indicate significant main effects of time [*F*_(1, 100)_ = 0.82, *p* = 0.365] or group [*F*_(2, 100)_ = 1.56, *p* = 0.215]. The group × time interaction was also not significant [*F*_(2, 135)_ = 2.44, *p* = 0.093].

For subtraction, no significant main effects of time [*F*_(1, 100)_ = 2.34, *p* = 0.129] or group [*F*_(2, 100)_ = 0.73, *p* = 0.486] were observed. There was a significant group × time interaction for subtraction [*F*_(2, 135)_ = 6.82, *p* = 0.002, η^2^ = 0.120]. The Calcularis group showed improvements in subtraction whereas the waiting group demonstrated a decreased performance in the computerized subtraction task [*F*_(1, 66)_ = 14.01, *p* < 0.001, η^2^ = 0.175]. The group x time interaction was not significant for the comparison between the Calcularis and the spelling training group [*F*_(1, 66)_ = 2.46, *p* = 0.122]. Additionally, there was a significant group x time interaction for the comparison between the spelling training group and the waiting group [*F*_(1, 68)_ = 4.55, *p* = 0.037, η^2^ = 0.06]. The spelling training group demonstrated an increase in performance while the children of the waiting group showed a decrease in performance.

#### Number line

##### Percent absolute error (PAE)

There were no significant main effects of time [*F*_(1, 87)_ = 1.32, *p* = 0.254] and group [*F*_(2, 87)_ = 2.44, *p* = 0.093]. The group × time interaction was significant [*F*_(2, 87)_ = 7.76, *p* = 0.001, η^2^ = 0.151]. Subsequent analyses indicated that the children of the Calcularis group demonstrated a significantly larger improvement than the children of the waiting group [*F*_(1, 59)_ = 15.04, *p* < 0.001, η^2^ = 0.203, large effect size] and the children of spelling training group [*F*_(1, 56)_ = 6.80, *p* = 0.012, η^2^ = 0.108, moderate effect size].

#### Linearity

The R^2^ lin was determined for each child individually. The GLM indicated that there was no main effect of time [*F*_(1, 87)_ = 0.03, *p* = 0.868] and no main effect of group [*F*_(2, 87)_ = 1.48, *p* = 0.233]. The group × time interaction was significant [*F*_(2, 87)_ = 6.85, *p* = 0.002, η^2^ = 0.136]. Subsequent analyses indicated that children of the Calcularis group demonstrated significantly stronger improvement than the children of the waiting group [*F*_(1, 59)_ = 10.99, *p* = 0.002, η^2^ = 0.157] with large effect size and the spelling training group [*F*_(1, 56)_ = 9.39, *p* = 0.003, η^2^ = 0.144] with large effect size.

Although there were no significant differences between the groups for age, we have to consider the large variation in age. Therefore, we re-analyzed the data using age as a covariate in the GLM. However, the results demonstrated that there were no substantial changes in the results of any group × time interaction.

Descriptive analyses of the feedback questionnaire demonstrated that the training was well received (*M* = 2.22, *SD* = 0.91, *n* = 41): 73.2% of the children indicated that they liked the training. Furthermore, 78.5% of the children reported an improvement of self-perceived arithmetic skills (*M* = 2.21, *SD* = 0.84) and 57.5% of the children indicated a better self-confidence (*M* = 1.68, *SD* = 1.12). 83.3% of the parents (20 of 24) reported that their child liked the training. The majority of the children rated the difficulty level of the training as appropriate (75.6%), with only few children perceiving the difficulty level as too high (7.3%) or too low (17.1%).

## Discussion

Several studies demonstrate that a notable proportion of children show insufficient basic knowledge of mathematics, which is predictive for further difficulties in learning mathematics (Jordan et al., 2003; Aubrey et al., 2006). Research on the development of numerical cognition and typical and atypical developmental trajectories is still in its infancy. The present article provides further insights into training approaches and mechanisms of action in order to enhance number processing and arithmetic skills at an early stage of math acquisition. Calcularis is an adaptive training software designed to support children in the math learning process. The program is based on a strong theoretical framework of numerical cognition and numerical development (Triple-Code-Model, Dehaene, 1992; four-step developmental model, von Aster and Shalev, 2007) and recent neuroscientific findings. The aim of this evaluative study was to examine whether the training program Calcularis is effective in enhancing arithmetic skills and spatial number representation. Our research design offers the possibility to compare the performance of the trained group with an untrained control group as well as to a group that received an alternative computerized training.

### Effects of the training program calcularis

#### Calcularis group vs. waiting group

The results are promising and showed significant improvements in half of the analyzed measures. This is in line with the results of a pilot study with a smaller sample (*N* = 32) of Swiss children with difficulties in learning mathematics (Käser et al., 2013). Compared to the waiting group, the Calcularis group demonstrated larger improvements especially with regard to subtraction with moderate to large effect sizes in all measures. This finding is regarded as solid benefit of the training since subtraction is considered as a strong indicator for the development of spatial number representation (Dehaene, 2011). Mental arithmetic like addition and in particular subtraction are facilitated by the growing mental number line. It facilitates not only mental backward and forward counting movements but also an efficient use of subtraction strategies like “indirect addition” (i.e., 42 − 37 can be changed into 37 + ? = 42), which requires a rapid estimation to decide if the two numbers are close enough to each other for indirect addition being an economic strategy (Kaufmann and von Aster, 2012; Linsen et al., 2014).

The results demonstrated no effects with regard to arithmetic performance measures for addition. To explain this finding, the hierarchical structure of Calcularis has to be considered. The training of addition/subtraction is carried out in ascending number ranges starting with the low number range 0–10. The next higher number range (0–10, 0–20, 0–100 etc.) is not unblocked before the child demonstrate arithmetic competencies (addition/subtraction) to a specific probability. Since the pre-test raw scores demonstrated that children performed better in addition than in subtraction, that the program provided in its adaptive design more training in subtraction leading to larger effects in subtraction than in addition.

Regarding spatial number processing two number line tasks with different number ranges (0–10, 0–100) were assessed to get more differentiating information of the improvements of spatial number representation since the program starts the training of mental number line tasks within the number range 0–10 and proceeds then to the number range 0–100. The Calcularis group showed stronger improvements in PAE and *R*^2^ lin than the waiting group in the computerized number line test ranging from 0 to 10 with large effect size. In the non-computerized number line test the Calcularis group demonstrated an increase in PAE within the number range 0–100, but this increase was not significantly higher than in the waiting group. Furthermore, the results indicate that over all three groups children demonstrate already a rather good linear spatial number representation in the 0–100 number range. This result is in line with former studies (e.g., Siegler and Booth, 2004; Kucian et al., 2011; Link et al., 2014). However, we did not find a significant improvement or group × time interaction for linearity in the 0–100 number range. Due to the hierarchical structure of the program, children are presented with a series of games that train spatial representation of numbers within the number range 0–10 and only continue to the next higher number range 0–100 when a definite accuracy is established. Therefore, these findings may suggest that solid training effects were obtained with regard to the number range 0–10 within the rather short training duration of 6–8 weeks, while more training is needed to establish a significant benefit to the spatial number representation within the higher number range 0–100. This is in line with Käser et al. (2013) who demonstrated that a prolongation of the training from 6 weeks to 3 months led to stronger effects, especially for the number line task within the number range 0–100. Nevertheless, these results show the beneficial effect of the program on the construction and access to the mental number line leading to an improved spatial representation of numbers.

This result is especially relevant as the formation of a mental number line constitutes a vital step in the numerical development (von Aster and Shalev, 2007) and studies demonstrated the significance of the mental number line for spatial number representation and arithmetic competencies (Siegler and Ramani, 2009; Kucian et al., 2011). However, it has to be considered that this improvement on the number line task might not only be due to an improvement of this underlying mental number line. The results of recent studies (Ashcraft and Moore, 2012; Hurst et al., 2014; Link et al., 2014; Peeters et al., 2016) indicate that the improvement could rather reflect an increasing use of helpful strategies like using reference points at the number line (e.g., imagining a midpoint on the number line).

Since studies that evaluate computerized training programs to enhance arithmetic performance or spatial number representation differ highly with regard to study samples (e.g., at risk learners or dyscalculic children) and targeted outcome measures, only a very limited amount of studies is available for adequate comparisons. Training studies demonstrating a high degree of comparability to our study, such as Lenhard et al. (2011) reported a moderate to large effect size for arithmetic performance for matched groups. Ise et al. (2012) analyzed the study of Fischer et al. (2008) and reported improvements in arithmetic skills with a large effect size after a daily training on subitizing and visual counting. Compared to these studies and the effect sizes reported in the meta-analyses of Kulik (1994), Li and Ma (2010), and Slavin and Lake (2008), training with Calcularis revealed considerable effect sizes: all effect sizes (of the significant group × time interactions) demonstrated medium to large sizes (η^2^ = 0.06–0.20). Furthermore, our sample demonstrated a high variance in arithmetic performance with low to high math performance levels (min: 1st percentile, max: 98th percentile). We expected the training effects therefore to be smaller than the effect sizes of studies with children at risk or with learning difficulties. Further analyses are needed to investigate who responds to the training and who may not and what factors are influencing the observed improvements.

#### Calcularis group vs. spelling training group

Compared to the spelling training group, the Calcularis group demonstrated stronger improvements in subtraction. In contrast to the findings of the Calcularis group compared to the waiting group, effect sizes are smaller. With regard to number line representation, children of the Calcularis group demonstrated improvements within the number range 0–10 (PAE, *R*^2^ lin) and 0 to 100 (PAE) with medium effect size compared to the spelling training group. Adequate comparisons to other studies are not possible, since most studies lack the comparison of training effects to a control group as well as a group of children receiving an alternative computerized training program. However, two promising studies can be taken into account: Obersteiner et al. (2013) as well as Fuchs et al. (2006). Obersteiner et al. (2013) used two modified versions of the program “Number Race” (Wilson et al., 2006) and compared the training groups to a control group that received a computerized language training program. They reported that the trained groups showed larger improvements than the control group with regard to arithmetical achievement with an effect size of *d* = 0.40. Since the design does not include an untrained control group, differentiated comparisons to our study results are not possible. Both training programs “Number Race” and “Calcularis” emphasize the mental number line and implemented number line tasks, but the comparability of “Number Race” and “Calcularis” is rather limited. “Number Race” focusses on numerical comparisons and trains number sense in kindergarten and preschool children, while “Calcularis” aims to automatize the different number representations and their interrelation and practice arithmetic operations as well as arithmetic fact knowledge in expanding number ranges in primary school children.

Fuchs et al. (2006) conducted a training to enhance arithmetic fact knowledge and compared the training group to a group that received a computerized spelling training. The authors report that the program was effective in promoting addition with large effect size (*d* = −0.82), whereas no results were found for subtraction or arithmetic story problems. Also in this study, the study design has no untrained control group implemented. With regard to these studies, reported effect sizes of the present study are comparable.

We expected smaller effect sizes for the comparison of the Calcularis with the spelling training group than for the comparison of the Calcularis with the waiting group. We assume that different cognitive as well as affective factors might be influenced leading to an improvement in the arithmetical outcome measures in both training groups (Calcularis and spelling training). However, the results indicated that children of the spelling training group did not increase more than the waiting group in almost all tasks, with the exception of the computer test subtraction. Nevertheless, we assume some relevant factors influencing training outcomes. First, the daily computerized training might have an effect on attention or working memory capacities. Therefore, the program influences superordinate cognitive functions that are crucial for information processing (e.g., learning, fact retrieval, problem solving), which might also be reflected in improved arithmetic performance. Second, the daily practice with the program might have an effect on affective variables (e.g., attitude, anxiety, self-concept), which might also contribute to the children's enhanced performance. There is extensive research demonstrating the relevance of attitude toward school subjects and the related academic achievement in the respective subjects (Ma and Kishor, 1997; Abu-Hilal, 2000). Furthermore, evaluative studies showed that computerized training programs have a beneficial impact on attitude toward school (Ke, 2008) and lead to increased motivation (Christmann and Badgett, 2003). Nevertheless, further research is necessary to uncover which aspects of computerized training programs might influence affective variables, especially motivation, and what role these affective changes play in relation to learning objectives and training effects.

In summary, to obtain a more complete picture of the single mechanisms of actions underlying an efficient computerized training, domain-specific and domain-general aspects of number processing and arithmetic as well as affective variables need to be taken into consideration (Kaufmann and von Aster, 2012).

Furthermore, when interpreting the results it has to be considered that there could be additional factors influencing the observed progression in the three groups that were not within the scope of this study. Conceivable are developmental factors that promote for instance a non-linear progression of development. These developmental changes may be not found immediately after the training, but later on.

The feedback questionnaire of the children and their parents revealed positive results, which correspond to the experiences and impressions of the study team during the training supervision sessions. This is especially significant as Calcularis is a learning system that is not embedded in a story game and has no reward system implemented. The majority of the children rated the general difficulty level of the training as appropriate. This serves as an indicator for the quality of the adaptation to the individual performance level and profile, the learning speed and the maintenance of the children's zone of proximal development (Vygotsky, 1978). Furthermore, in-depth analyses of the children‘s path through the skill net illustrated that children show highly diverse profiles of mathematical knowledge and deficits leading to very different training trajectories which optimize the learning process (Käser et al., 2012, 2013). These findings highlight the need of adaptation to the child‘s individual knowledge level on the one hand and prove the efficiency and adaptability of the student model and the control algorithm on the other.

### Limitations and further research indications

Several important limitations of this study should be noted. First, Calcularis combines the training of basic numerical skills, spatial number representations and arithmetic operations. The evaluative data revealed good effects for subtraction and spatial representation of numbers. However, basic numerical skills (e.g., number/quantity comparisons, subitizing) were not assessed. Therefore, training effects with regard to basic numerical skills could not be determined. In addition, there was no assessment of the children's performance in multiplication and division which would have served as a measures for training transfer. Second, the discriminate training transfer could not be evaluated since we did not assess non-numerical measures (e.g., reading, spelling) after the training. Third, the reported results respond to immediate efficacy. Therefore, the present results do not allow for conclusions about increased mathematical competencies or transfer to other arithmetic performances in the long-term. Fourth, in the present study more than two thirds of the study population were girls, but gender ratio deviated not significantly over the groups. Participants were recruited through advertisements distributed to professionals and schools. Therefore, the offer of a computerized training program for enhancing numerical cognition showed to be especially attractive to girls. Future research with larger samples and equal gender ratios is needed for more differentiating analyses of possible effects of gender. Following this first evaluation stage of Calcularis, a further evaluative study is currently conducted that includes the implementation of a control group receiving conventional integrative learning therapy for children with developmental dyscalculia. Thus, we aim to understand the underlying processes and mechanisms of action of a computerized training program compared to non-computerized learning approaches.

## Conclusion

This study demonstrates that the adaptive training program Calcularis can be used effectively to support children in their numerical development and to enhance subtraction and spatial number representation. While other computerized training programs revealed good training effects for arithmetic performance (e.g., Fischer et al., 2008; Lenhard et al., 2011) or spatial number representation (e.g., Kucian et al., 2011), Calcularis demonstrated even after a rather short training period (6–8 weeks) good effects for both. The article raises questions about the general mode of action of computerized programs. Future research is necessary to uncover, which cognitive, affective and motivational variables are affected in computerized programs leading to enhanced arithmetic performance. Finally, it should be noted, that Calcularis was conceptualized to support learning processes. We completely agree with Kozma (2001) who argues that there is no tool (computerized programs etc.) that will replace good teaching. The Calcularis training program does not aim to replace conventional teaching methods, but it might be a valuable opportunity to supplement and support teachers and to create a more differentiating and inclusive teaching environment.

## Author contributions

GE, KK, MV, and JK conceived the study. GE, KK, MV, JK, UM, VM, and TK designed the study. JK, LR, VM, KK, TK, and UM performed research. JK, LR, and MV analyzed the data. JK and LR wrote the manuscript.

## Funding

This work was supported by research grant 01GJ1011 from the German Federal Ministry of Education and Research (BMBF).

### Conflict of interest statement

The authors declare that the research was conducted in the absence of any commercial or financial relationships that could be construed as a potential conflict of interest.
